# Enhancement of wind energy conversion system performance using adaptive fractional order PI blade angle controller

**DOI:** 10.1016/j.heliyon.2021.e08239

**Published:** 2021-10-22

**Authors:** Ahmed M. Shawqran, Abdallah El-Marhomy, Mahmoud A. Attia, Almoataz Y. Abdelaziz, Hassan Haes Alhelou

**Affiliations:** a Physics and Engineering Mathematics, Faculty of Engineering, Ain Shams University, Cairo, Egypt; b Electrical Power and Machines, Faculty of Engineering, Ain Shams University, Cairo, Egypt; c Faculty of Engineering and Technology, Future University in Egypt, Cairo, Egypt; d School of Electrical and Electronic Engineering, University College Dublin, Ireland

**Keywords:** Wind energy, Blade angle controller, Fractional calculus, Adaptive PI, Adaptive fractional order PI, Equilibrium optimization EO

## Abstract

Wind energy is considered as one of the rapidest rising renewable energy systems. Thus, in this paper the wind energy performance is enhanced through using a new adaptive fractional order PI (AFOPI) blade angle controller. The AFOPI controller is based on the fractional calculus that assigns both the integrator order and the fractional gain. The initialization of the controller parameters and the integrator order are optimized using the Harmony search algorithm (HSA) hybrid Equilibrium optimization algorithm (EO). Then, the controller gains ($K_{p},\;K_{i}$) are auto-tuned. The validation of the new proposed controller is carried out through comparison with the traditional PID and the Adaptive PI controllers under normal and fault conditions. The fractional adaptive PI improved the wind turbine's electrical and mechanical behaviors. The adaptive fractional order PI controller has been subjected to other high variation wind speed profiles to prove its robustness. The controller showed robustness to the variations in wind speed profile and the nonlinearity of the system. Also, the proposed controller (AFOPI) assured continuous wind power generation under these sharp variations. Moreover, the active power statistical analysis of the AFOPI showed increase in energy captured of around 25 %, and reduction in the standard deviation and root mean square error of around 10% compared to the other controllers.

## 1 Introduction

**Figure 1:**
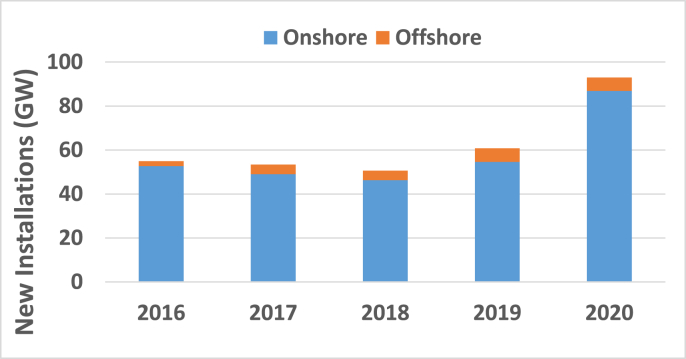
New installations of the wind technology all over the world by GWEC.

The increased population census forced the world to increase investments in improving sustainable energy sources. The most currently popular sources of renewable energy are solar energy, wind energy, hydro energy, and tidal energy. But, renewable energy affected the power system performance, which is considered a challenge to the researchers [1]. Wind is a plentiful source of clean energy. Also, it is one of the rapidest-rising renewable energy technologies according to the Global Wind Energy Council (GWEC). The global wind power industry's record year was 2020 [1, 2]. This year witnessed 93 GW wind power installations divided into 86.9 GW onshore and 6.1 GW offshore, which is considered an increase in the global wind power percentage by around 53% compared to 2019. Also, it is considered an increase in the total capacity by 14% than 2019 capacity to be 743 GW as shown in Figure 1 [3]. Much research has been applied to enhance the performance of wind farms to keep-up with this obvious wind energy installation growth. Thus, the paper proposed a new blade angle controller to achieve this target.

**Figure 2:**
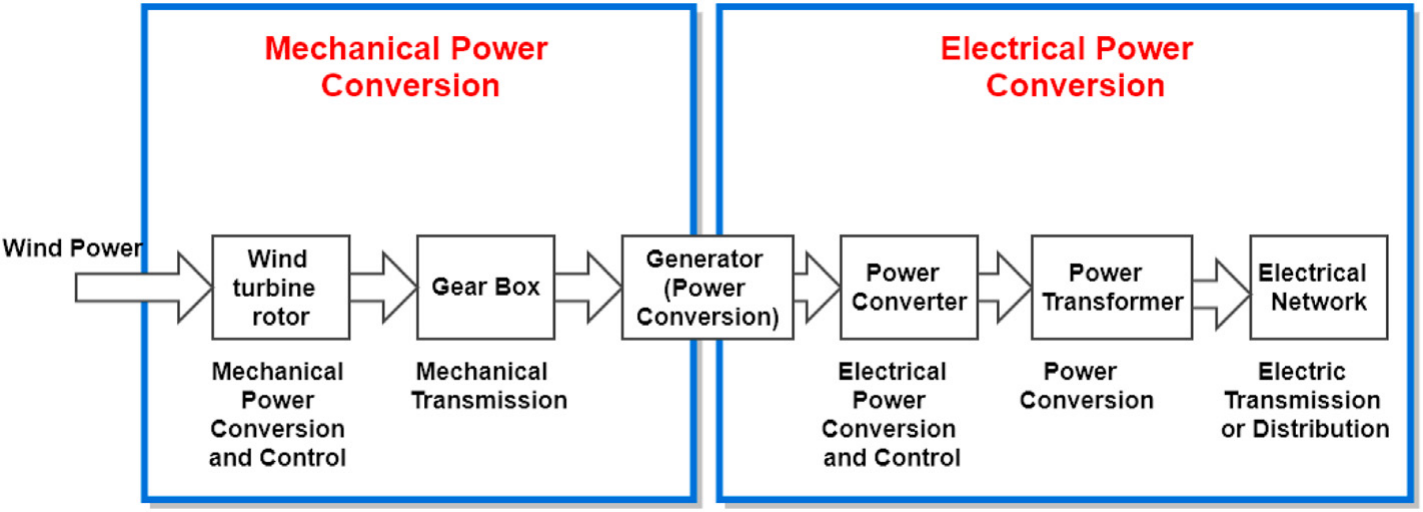
The wind turbine system conversion systems.

The wind energy of conversion system (WECS) converts the power from one form to another. It consists mainly of two main stages: mechanical power conversion and electrical power conversion as shown in Figure 2. The first stage converts the aerodynamic power stored inside the wind into mechanical power inside the rotational blades. The mechanical parameters that can be controlled in this stage are generator rotor speed, gear box ratio, yaw angles, and blade angles. The second stage converts the rotational mechanical power into electrical power through synchronous and asynchronous generators connected directly to the gird or through power converters [4]. The electrical controlled parameters are active power, output voltage, reactive power, and frequency.

**Figure 3:**
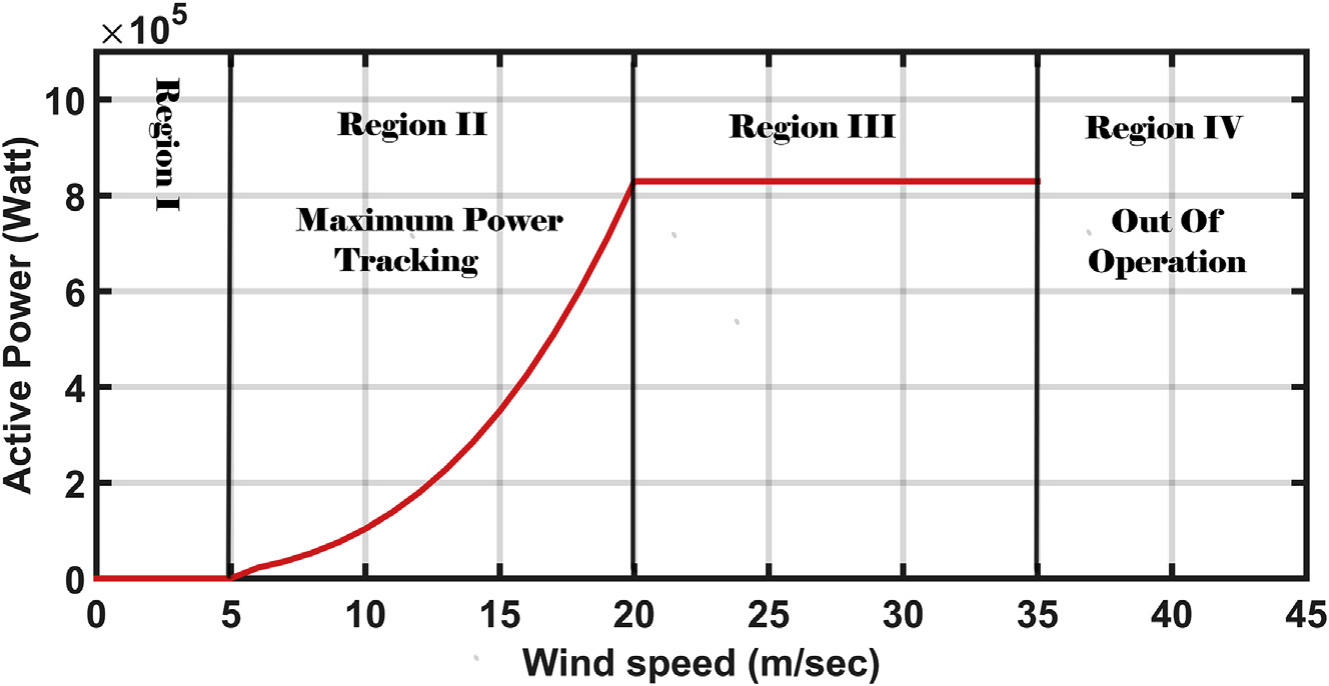
The four control regions of the wind turbine.

The most effective controller in the mechanical power conversion stage is the blade angle controller. This controller adjusts the blade angles to regulate the aerodynamic power of the turbine system. According to the power-speed curve shown in Figure 3, there are four regions of operation [5]. The second and third regions are the only controllable regions. In the second region, maximum power point tracking is carried out. While in the third region, the blade angle controller is operated to keep the power at its rated value under gusty wind speeds.

The wind turbine pitch angles are controlled by several techniques such as the proportional-integral-derivative (PID) and PI [6]. They are widely applied due to its advantages. While, they are simply designed controllers. But their major disadvantage is their operation specific limits. These limits are determined by the system nonlinearity.

In last decades, the wind turbine blade angle is controlled by new techniques such as: adaptive controllers, fuzzy logic, and sliding mode. The sliding mode controller is considered as one of the optimum nonlinear controls. Its nonlinearity properties promote it to be used widely in wind turbine systems [7]. The sliding mode controller was developed using a second-order linear system first. Then it was extendedly applied to other systems such as: multi variable and discrete systems. For complex systems, the sliding mode controller succeeded in controlling them with good reliability. It enhances system robustness while solving the uncertainties in the wind system parameters through increasing the robustness of its dynamic responses.

While fuzzy logic is considered as a supreme method to control wind turbine blade angles due to the fluctuations in the wind speed profiles, which affect many factors such as: wind system dynamics, mechanical torque, and the speed of the generator rotor. The linguistic variables are formed by converting the wind system errors into fuzzy codes. These errors include the error of the rotor speed and the error of the numerical [8]. The fuzzy linguistic variables can adapt themselves to track up any fluctuations in the wind turbine system parameters through acquiring novel methods of learning, which is the main merit of the fuzzy controllers. This advantage makes fuzzy logic controllers suitable for the variation of the wind speed profile that occur in an unpredicted sudden mode throughout the four seasons of the year. Authors of [9, 10] carried out many comparisons between the traditional PID and the fuzzy controllers, to prove the distinction of the fuzzy controllers.

Nowadays, many trials have been carried out to improve the PID controller performance by using fractional-order controllers [11, 12] based on fractional calculus. The Fractional order PID has two additional tuning parameters than the traditional PID controller, resulting in improved closed-loop performances and system robustness.

The performance of the controllers relies mainly on the values of the parameters. So, to enhance the controller behavior various techniques are applied to tune the controller parameters such as: artificial intelligence, and optimization algorithms. The genetic optimization algorithms (GA), the search of harmony optimization algorithm, and the learning-based teaching optimization algorithm (TLBO) are the most applied techniques in tunning the controllers parameters [13, 14, 15].

Relying on the self-adjusting gains, the distinction of the classical adaptive PI over the PID was proved. These self -adjusting gains can track up the variations of the system parameters. In this paper, an adaptive fractional order PI pitch angle controller is presented and evaluated. The new proposed controller acquires the advantage of both the conventional adaptive PI, which is the self-adjusted gains and the advantage of the fractional order integrator. The sequence of the paper is divided as follows: in the first section, the model equations of the wind turbine system and the aerodynamics of the turbine blade are presented. The second section explains the equations of the proposed adaptive fractional order proportional integrator (AFOPI) controller. The third section illustrates the methodologies and the flow charts of the different optimization algorithms. In the fourth section, the controller validation is held through comparison with the conventional adaptive PI and classical PID under normal and fault conditions. In the fifth section, different wind speed profiles are applied to the new proposed adaptive controller to assure its robustness. The Last section discusses the contribution and conclusion.

## 2 Wind turbine modelling equations

The wind turbine generates the mechanical energy from the energy stored inside the upcoming wind. The mechanical power is directly proportional to the cube of the velocity and can be explained as:

(1) $$P_{m}=\frac{1}{2}\rho AC_{p}\left(\lambda ,\beta \right)V_{\omega }^{3}$$

where ρ, *C*_*p*_(λ,β), $V_{\omega }$, A, and β are the density of the air, the power coefficient of the wind-turbine (Betz limit constant), the wind profile velocity, the rotor blades swept area, and the blade pitch angle respectively [16]. In 1919 a German physicist called Albert Betz assured that the maximum efficiency $\left(C_{p}\right)$ can ideally reach 0.59 value [17], and can be given as:

(2) $$C_{p}=\;C_{1}\left(\;\frac{C_{2}}{\lambda_{i}}-C_{3}\beta -C_{4}\right)e^{\frac{-C_{5}}{\lambda_{i}}}+C_{6}\lambda$$

(3) $$\frac{1}{\lambda_{i}}=\left(\frac{1}{\lambda +0.08\beta }-\frac{0.035}{\beta^{3}+1}\right)$$

Where *C*_1_, *C*_2_, *C*_3_, *C*_4_, *C*_5_, and *C*_6_ are the power coefficient constants listed in Table 1.

**Table 1:** Turbine geometrical data.

Nominal mechanical output power	1.5e6 watt
Minimum pitch angle	0 deg
Maximum pitch angle	45 deg
Maximum rate of change of pitch angle	2 deg/s
*C*_1_, *C*_2_, *C*_3_, *C*_4_, *C*_5_, *C*_6_	0.51763, 116, 0.4, 5, 21, 0.006795
$C_{p\_nominal}$	0.48
$\lambda_{nominal}$ (tip speed ratio)	8.1
Base wind speed	12 m/s
Inertia constant, friction factor, and pairs of poles	0.5, 0.01, 3
Blade length	8.1 m
Base rotational speed (pu of base generator speed)	1.2 pu

The rotational velocity of the blades of the rotor is given by ω (in rad/sec), the linear speed varies for each point along the length of the blade. The tip speed ratio (TSR) describes this speed variation as follows:

(4) $$TSR=\lambda =\frac{R\;\omega }{V_{\omega }}$$

where R is the distance from the rotation axis to the blade tip. The maximum power at every wind speed is achieved by acquiring the optimum TSR. This supreme TSR is achieved at a certain angle of attack (supreme angle of attack). A design tip speed ratio could be chosen which corresponds to the wind speed at the specified site which contains the most energy. The key role in the power equations is the blades' angles. They control the angle of attack, which in turn controls the lift and drag forces of the blade.

**Figure 4:**
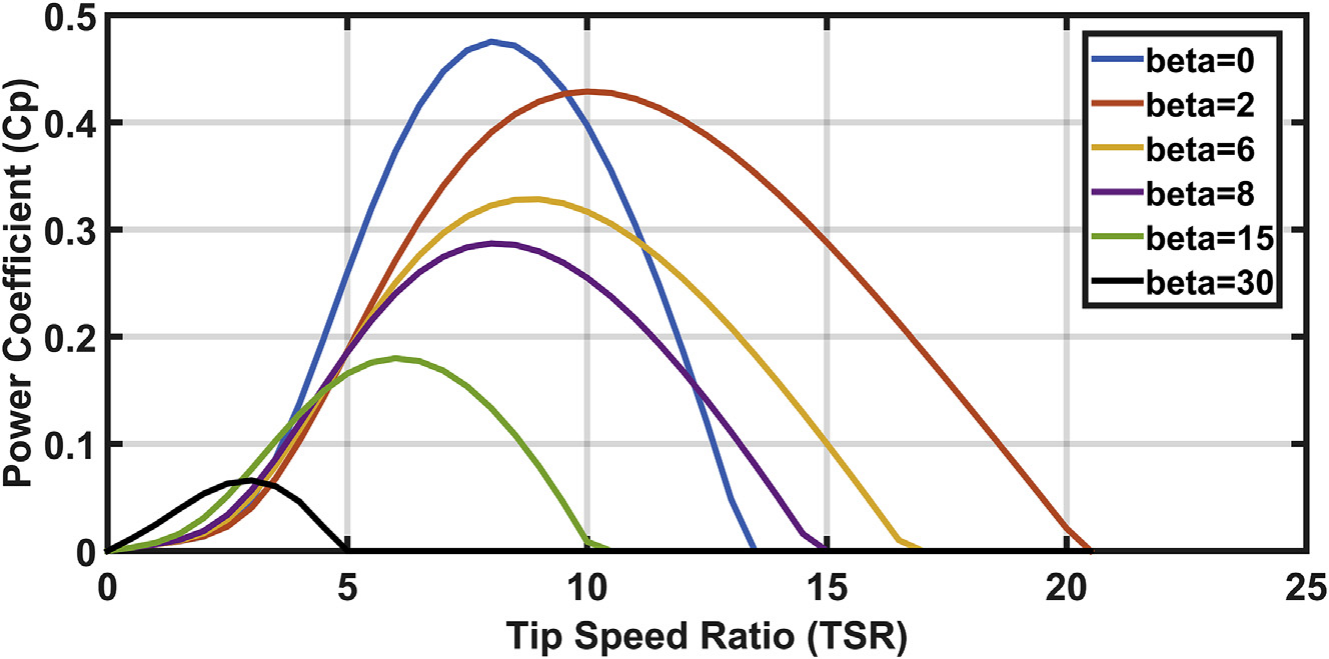
The conversion coefficient of power versus tip speed ratio (Cp – λ) curve for different blade pitch angle (β°).

Figure 4 depicts the effects of blade angle variations on the power coefficient and tip speed ratio. The maximum extracted power is achieved at zero blade angle and nominal lambda. Moreover, Figure 4 shows the influence of blade angle changes on the blade velocity, which in turn controls the tip speed ratio [18].

According to operation, wind turbines are mainly divided into four main categories:

• Fixed speed fixed pitch (FS-FP):

In this scheme, the asynchronous squirrel-cage induction generator (SCIG) generator is directly connected to the grid. Fixed-speed operation indicates that maximum power is achieved only at a single wind speed. The turbine is designed to operate at a fixed pitch angle. Thus, at low speeds, power is below the rated power, power limitation is achieved by passive stall, and conversion efficiency cannot be optimized. while at high wind speeds, FS-FP wind turbines are stall regulated.

• Fixed-speed Variable-pitch (FS-VP):

Variable pitch indicates that the blades are freely allowed to rotate about their axis, so they are attached to the WT hub through gears and servo motors. In low wind speeds, variable-pitch operation can enhance energy capture. In above the rated wind speeds, power is limited by continuously adjusting the pitch angle. There are basically two methods of power regulation by pitch control, namely pitch-to-feather and pitch-to-stall.

• Variable-speed Fixed-pitch (VS-FP):

In variable speed operation, frequency converters are interposed between the generator and the network. Doubly fed of induction generators are installed, which can operate at different rotor speeds. The stator winding of the DFIG is directly connected to the grid, while the rotor winding is connected to power converters of rating approximately up to 30% of the rated power. In VS-FP, the blades are promptly attached to the wind turbine hub, so the blades are directed at a certain constant angle. This blade fixation allows the tip speed ratio to be changed in a very narrow range.

• Variable-speed Variable-pitch (VS-VP):

For low power, the blades can be turned into the wind, while for high power the blades are turned away from the wind. So, the turbine nearly operates at variable speed and fixed pitch below rated wind speed and at variable pitch above rated wind speed. Variable-pitch turbines allow blade angle controllers to optimize the extracted power below the rated speed and to regulate power above the rated wind speeds [19].

**Figure 5:**
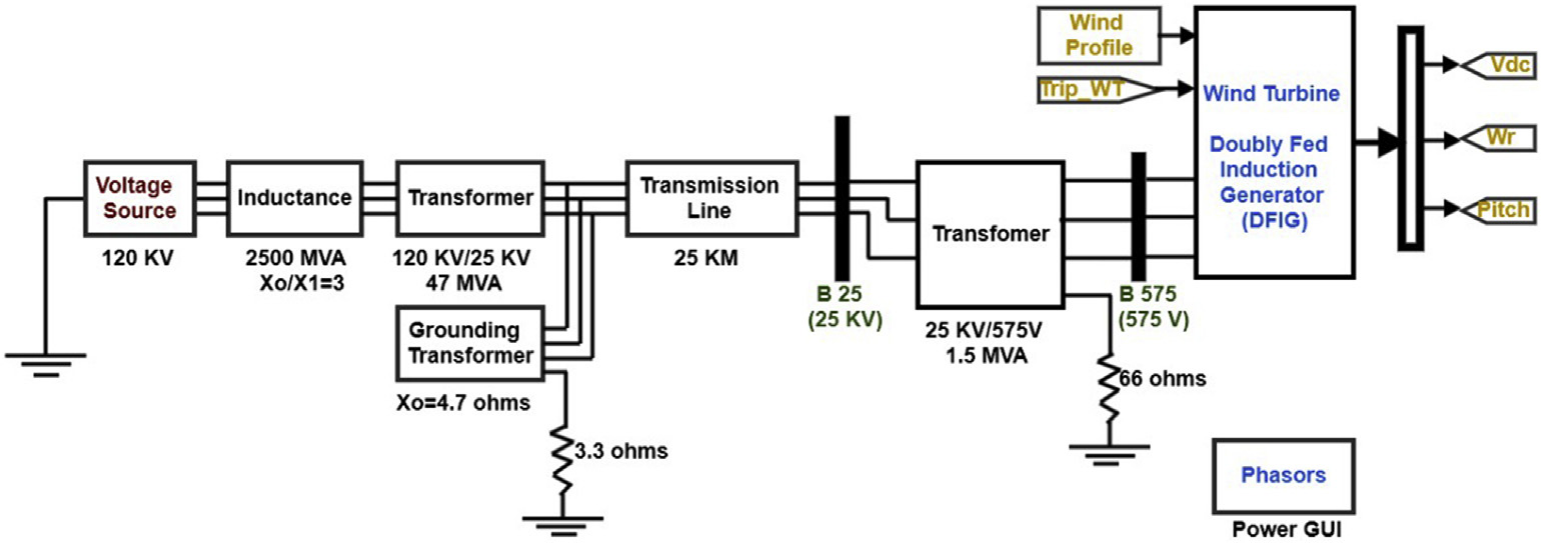
The wind turbine DFIG MATLAB representation model.

Figure 5 shows the wind farm MATLAB model representation. This wind farm has rating of 9 MW and is connected to the grid.

The geometrical turbine data is listed in Table 1.

## 3 Blade pitch angle controller

### 3.1 Adaptive PI (API) controller

The conventional PI controller consist of two gains: proportional gain (Kp) and integral gain (Ki) as given in Eq. (5). Authors of [20, 21] updated the conventional PI controller to be adaptive as illustrated in Eqs. (6), (7), (8), and (9).

(5) $$\text{C}\left(\text{s}\right)=K_{p}+\left(K_{i}/s\right)$$

(6) $$Output\left(t\right)=-K_{c}\left(error*error+\int_{0}^{t}error*error\;dt\right)$$

Where *K*_*p*_, *K*_*c*_, *K*_*i*_, and e(t) are the proportional gain, the constant gain, the integral gain, and the error signal respectively.

(7) $$error=\omega \;-\;\omega_{ref}$$

Where $\omega_{ref}\;$is the reference of the generator rotor speed.

(8) $$K_{p}=error*error+K_{1}\int_{0}^{t}error*error\;dt$$

(9) $$K_{i}=K_{2}\int_{0}^{t}error*error\;dt$$

**Figure 6:**
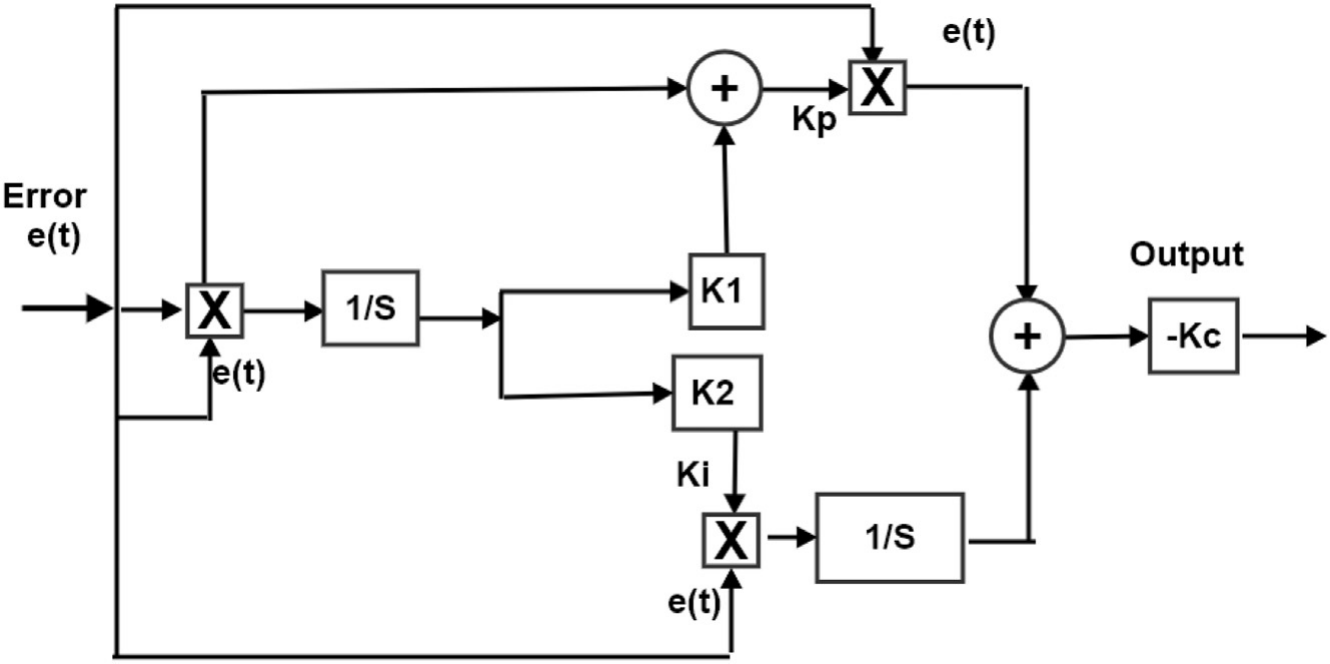
The representation of the block diagram of the adaptive PI controller.

Where *K*_1_ and *K*_2_ are the adaptive initialization parameters [20, 21, 22]. Figure 6 illustrates the adaptive controller operation, the proportional (*K*_*p*_) signal, and the integral (*K*_*i*_) signal. The *K*_p_ and *K*_*i*_ are auto-adjusted.

### 3.2 Adaptive fractional order PI (AFOPI) controller

The idea of the adaptive fractional order PI (AFOPI) depends mainly on the adaptive PI in the previous section and a field of mathematics called Fractional calculus (FC). The fractional calculus assigns an arbitrary fractional order to derivatives and integrals terms. These fractional-order systems can be described by a Linear Time Invariant (LTI) fractional-order differential equation [23, 24, 25]. The traditional fractional order transfer function is given in Eq. (10). In the same manner as the previous controller, the AFOPI controller equations can be deduced from Eqs. (5), (6), (7), (8), (9), and (10). Eqs. (11), (12), and (13) presented the AFOPI controller.

(10) $$\text{C}\left(\text{s}\right){=\text{K}}_{\text{p}}\left(\text{K}\right)+\left({\text{K}}_{\text{i}}/{\text{s}}^{\text{m}}\right)$$

(11) $$K_{p}=error*error+K_{1}\int_{0}^{t}error*error\;dt$$

(12) $$K_{i}=K_{2}\int_{0}^{t}error*error\;dt$$

(13) $$Output=-K_{c}\left(K_{3}K_{p}\left(t\right)error+\left(1/S^{m}\right)K_{i}\left(t\right)error\right)$$

**Figure 7:**
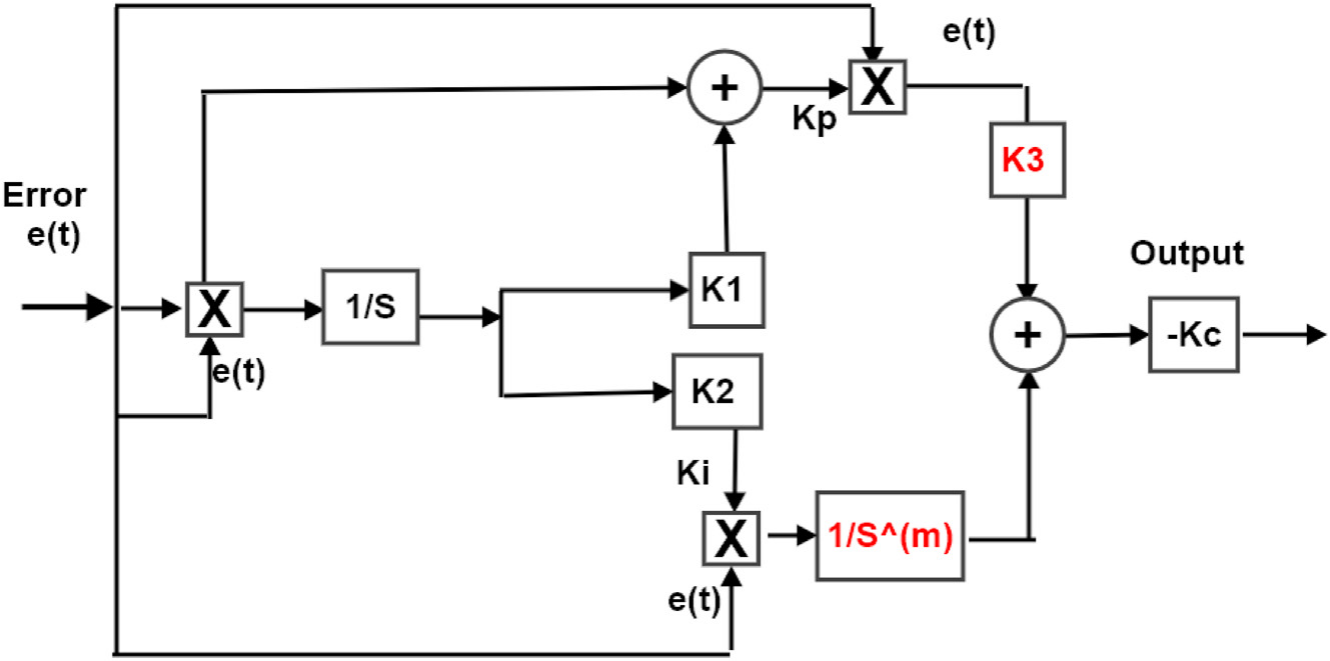
The representation of the block diagram of the AFOPI controller.

Where *K*_3_ is a fractional constant value, and m is the order of the fractional integrator. Both *K*_3_ and m range from zero to one. The block diagram of the AFOPI is shown in Figure 7.

## 4 Techniques of optimization algorithms

The behavior of the system can be upgraded by initializing the controller parameters. This parameter initializations can be carried out through the application of different optimization algorithms. The per unit difference between the voltage reference and actual voltage (ΔV) is summed up with the per unit difference between the power reference and actual power (ΔP). This summation is the optimization objective function which should be minimized. The optimization algorithms select the initial values of the adaptive PI (API) and adaptive fractional order PI (AFOPI) then the adaptive controllers auto-tune their gains. The explanation of the algorithms is in the next sections:

### 4.1 Harmony search algorithm HSA

**Figure 8:**
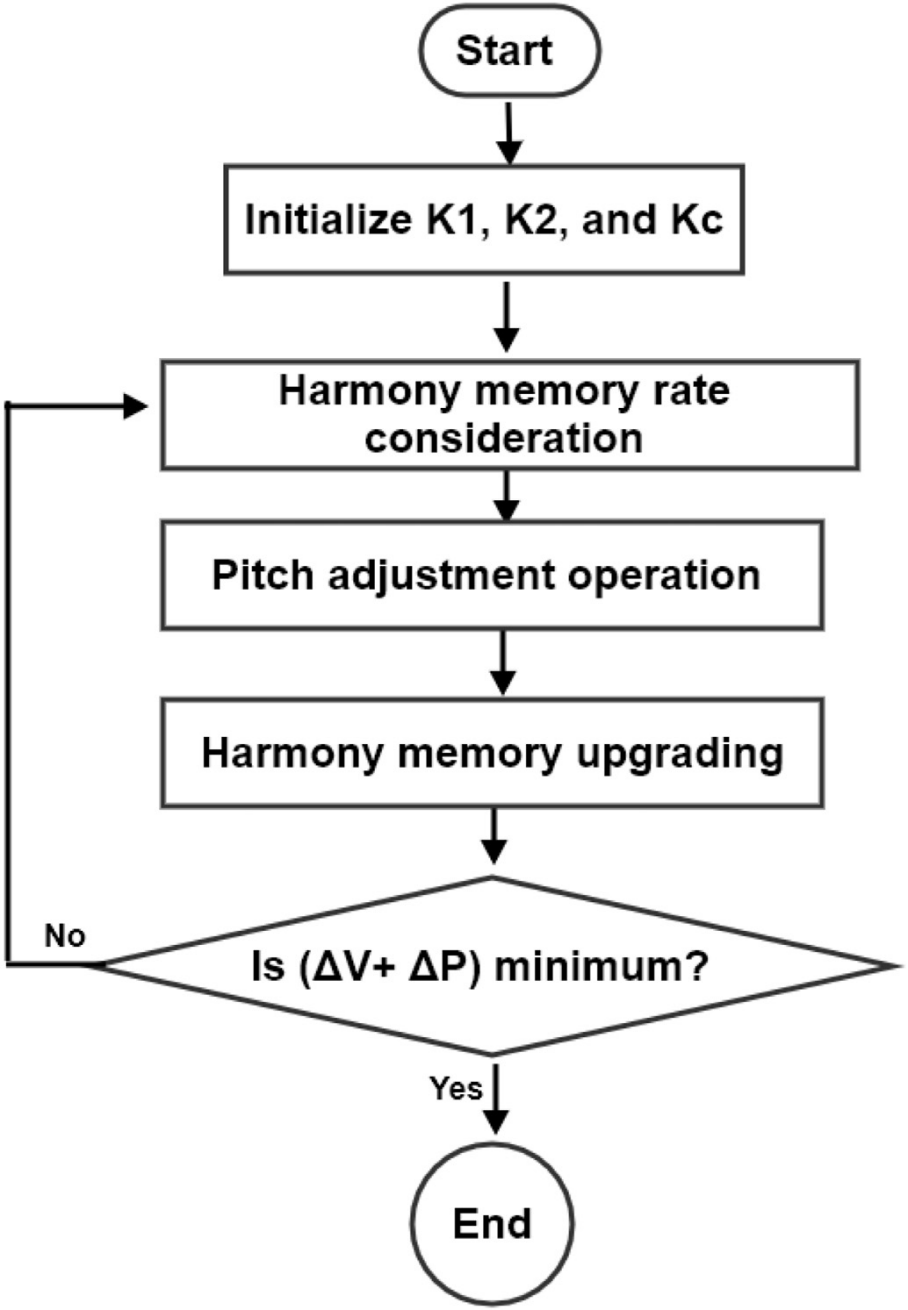
The representation of the flow diagram of the HSA algorithm.

The HSA was inspired by the musical performance process. The algorithm is made up of three operators: random search, harmony memory considering rule, and pitch adjusting rule [26]. These three operators determine the ways of handling exploration and exploitation which make the HSA a unique metaheuristic algorithm. Three basic stages determine the harmony search algorithm implementation: harmony memory rate consideration, pitch operation adjustment, and harmony memory upgrading as shown in the flow diagram in Figure 8.

### 4.2 Genetic algorithm GA

**Figure 9:**
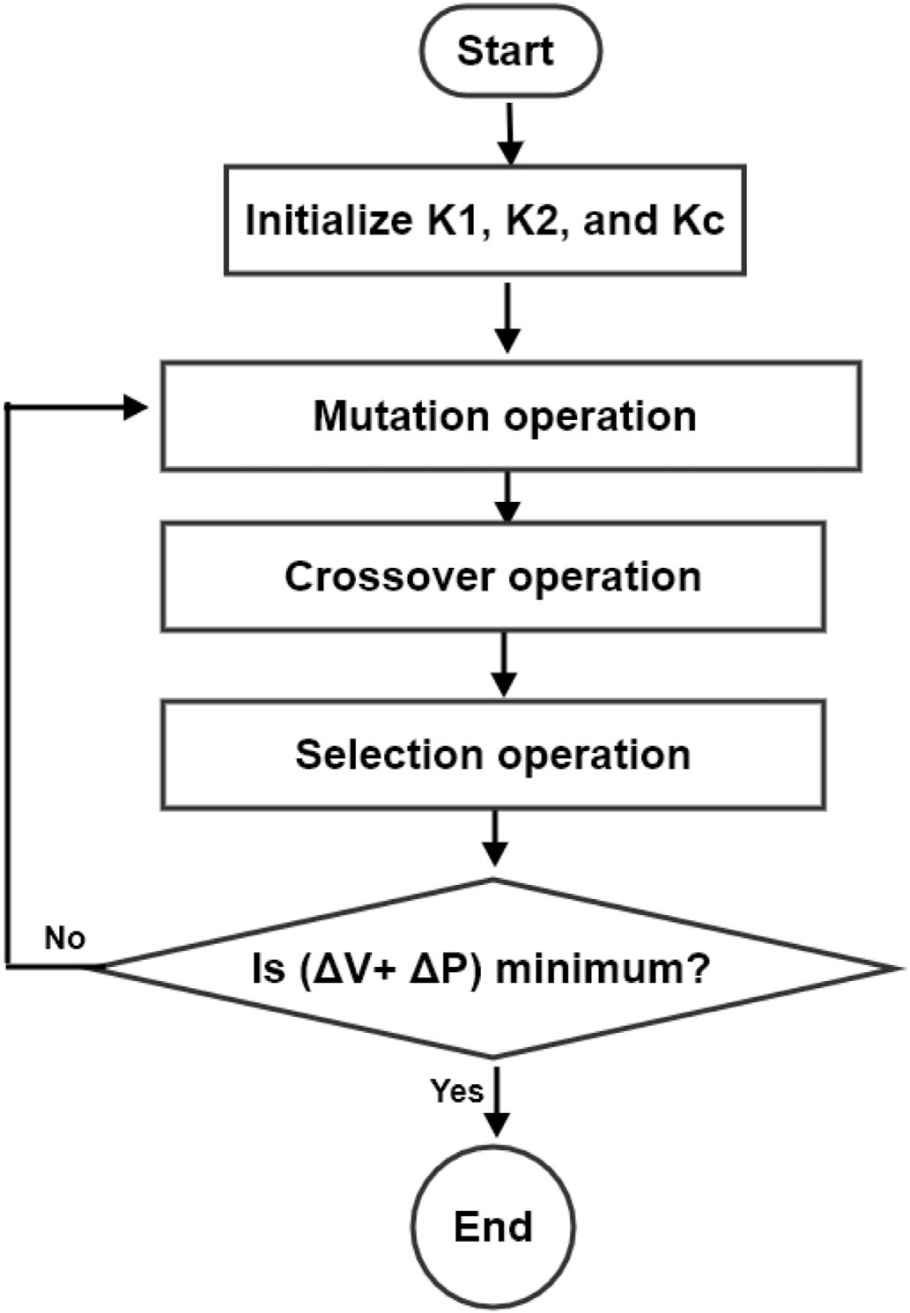
The representation of the flow diagram of the GA algorithm.

Depending on the natural selection process, the genetic algorithm (GA) succeeded in solving both constrained and unconstrained optimization problems. These natural processes mimics biological evolution. Genes are the only factors that make winner success which are spread by the reproduction of such individuals. The population will obtain more natural resources by successive selection of superior individuals and reproducing them [27]. The genetic algorithm sequence procedure is shown in the flow diagram in Figure 9.

### 4.3 Teaching learning based optimization TLBO

**Figure 10:**
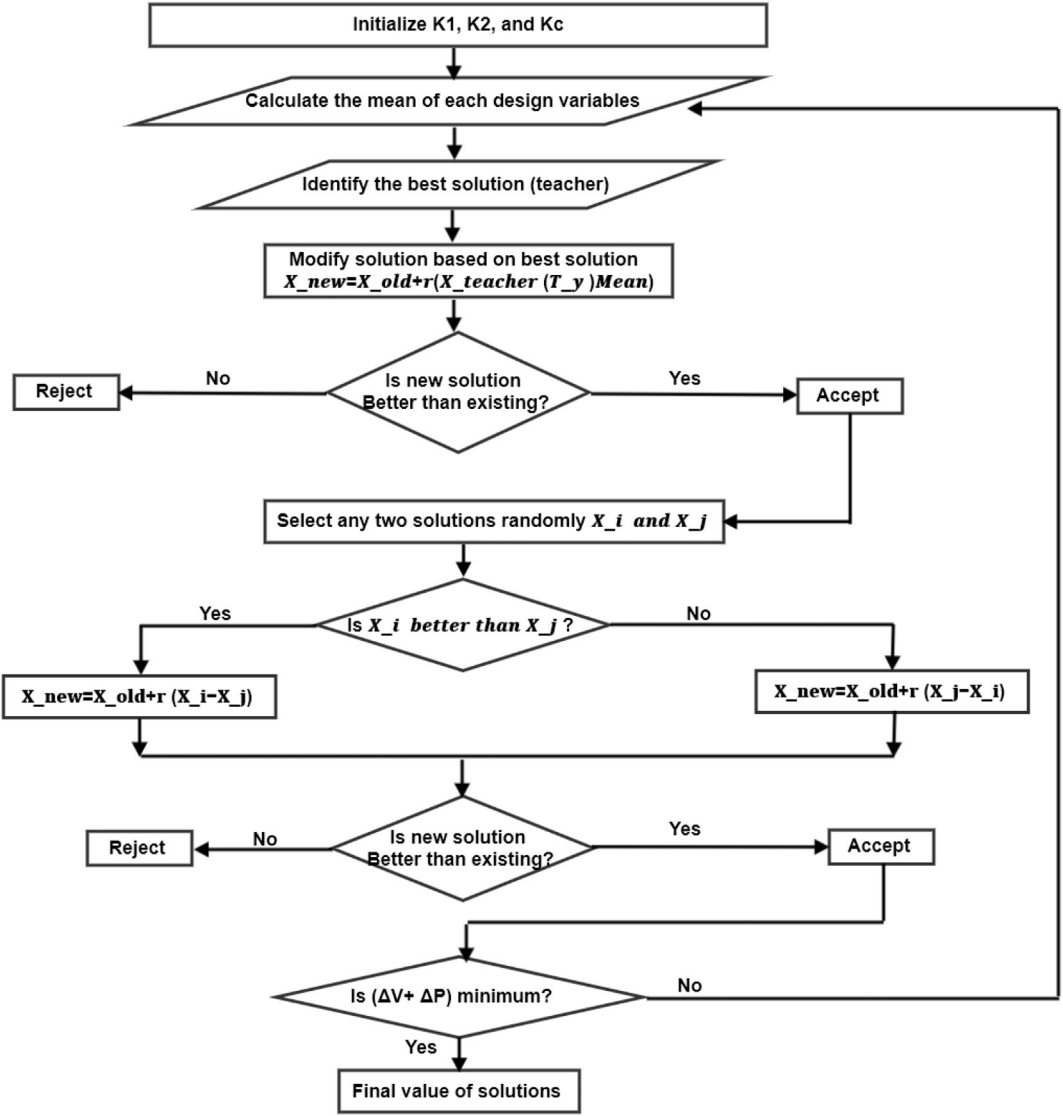
The representation of the flow diagram of the TLBO algorithm.

TLBO has two main influences: the influence between the teacher and learners and the influence between the learners themselves [28]. The implementation of the TLBO algorithm occurs in five steps as shown in the flow diagram in Figure 10.

### 4.4 Equilibrium optimization EO

**Figure 11:**
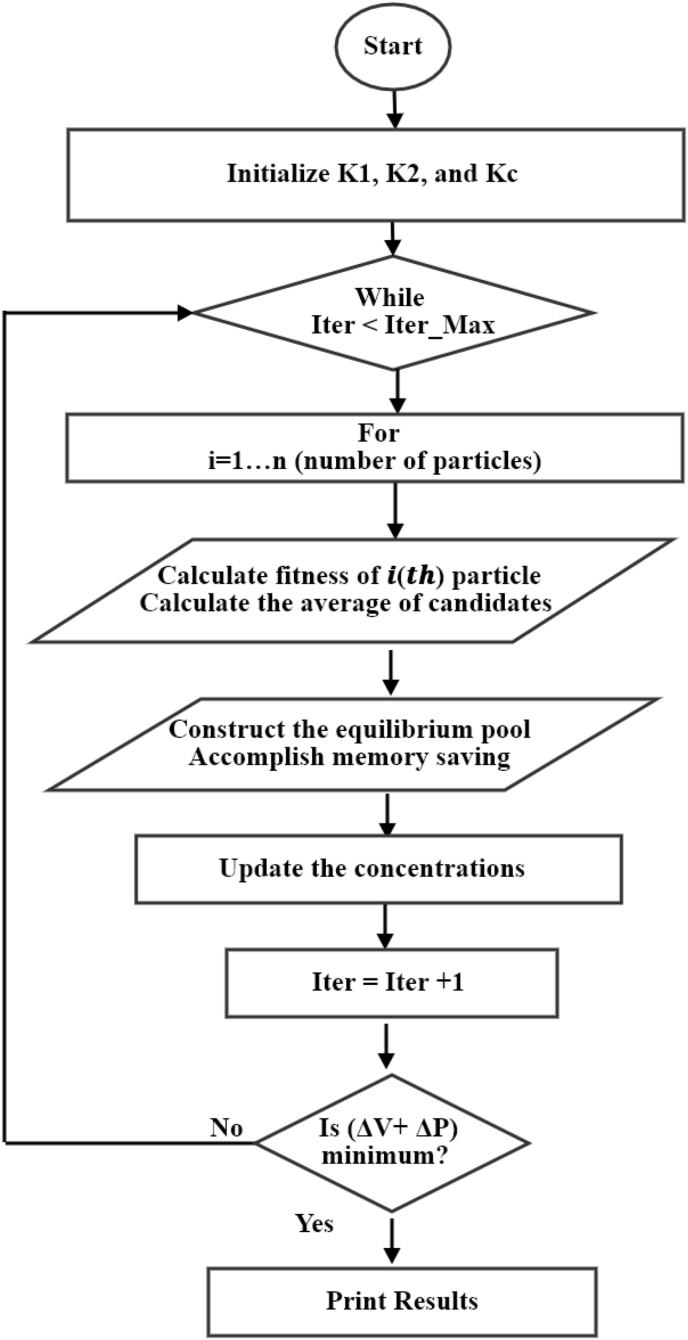
The representation of the flow diagram of the EO algorithm.

The Equilibrium Optimizer (EO) is a novel optimization algorithm where both dynamic and equilibrium states can be estimated by the mass equilibrium equations. The mass equilibrium equation provides the underlying physics for the conservation of mass entering, leaving, and generated in a control volume. The optimization process is started by the initial population, which is used in many meta-heuristic algorithms. Based on the number of particles and dimensions, the EO constructs the initial concentrations. The optimization steps of the EO are listed in the flow diagram shown in Figure 11 [29].

### 4.5 HSA hybrid GA (HSA-GA)

Combining the HSA algorithm with the GA algorithm adds more enhancement to the system performance. So, hybridizing different search strategies can promote a more enhanced algorithm. The HSA algorithm is applied firstly, and the system optimum solution is determined. Then, the lower and the upper boundaries of the GA parameters are settled with a ±10 % tolerance of this optimum solution [30].

### 4.6 HSA hybrid TLBO (HSA-TLBO)

Other hybrid combination technique is to apply the HSA algorithm firstly to determine the system optimum solution. Then, the lower and the upper boundaries of the TLBO parameters are settled with a ±10 % tolerance of this optimum solution [24].

### 4.7 HSA hybrid EO (HSA-EO)

The proposed hybrid system is to use the HSA algorithm firstly to system best solution, then this best solution is used to set the upper and lower boundaries of EO parameters with a ±10 % tolerance.

## 5 Validation of the AFOPI controller

A comparison is held between the proposed adaptive fractional order PI (AFOPI), the conventional PID, and adaptive PI under normal and fault conditions. This comparison assures the distinction of the proposed controller over the other controllers.

### 5.1 Validation under normal conditions

**Figure 12:**
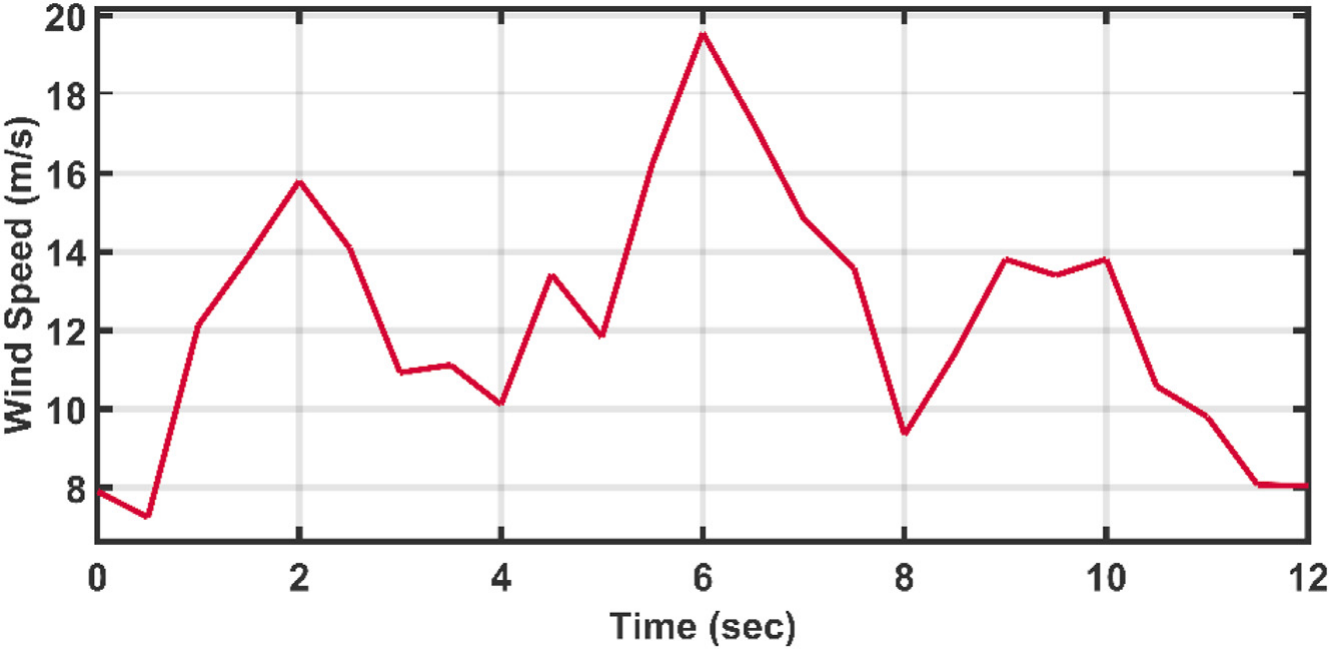
The speed profile of the applied wind.

Figure 12 shows the applied profile of winter wind speed to the wind turbine. The initializations of the API are carried out by using HSA-GA and HSA-TLBO [30], while the AFOPI initializations are carried out by using HSA-EO and the gains of the PID are tuned with the HSA optimization algorithm [31]. The parameters of the AFOPI, API, and traditional PID gains are listed in Table 2.

**Table 2:** The controllers parameters.

Controllers	Controller gains		
PID [31]	***K***_***p***_	***K***_***i***_	***K***_***d***_
	68	13.7	72
	Controller Parameters		
	**Kc**	**K**_**1**_	**K**_**2**_
API HSA-GA [30]	24	132	743
API HSA-TLBO [30]	26.63	155.25	817.54
AFOPI HSA-EO	16.11	32.54	1115.4
	m	***K***_**3**_	
	0.83	0.155	

**Figure 13:**
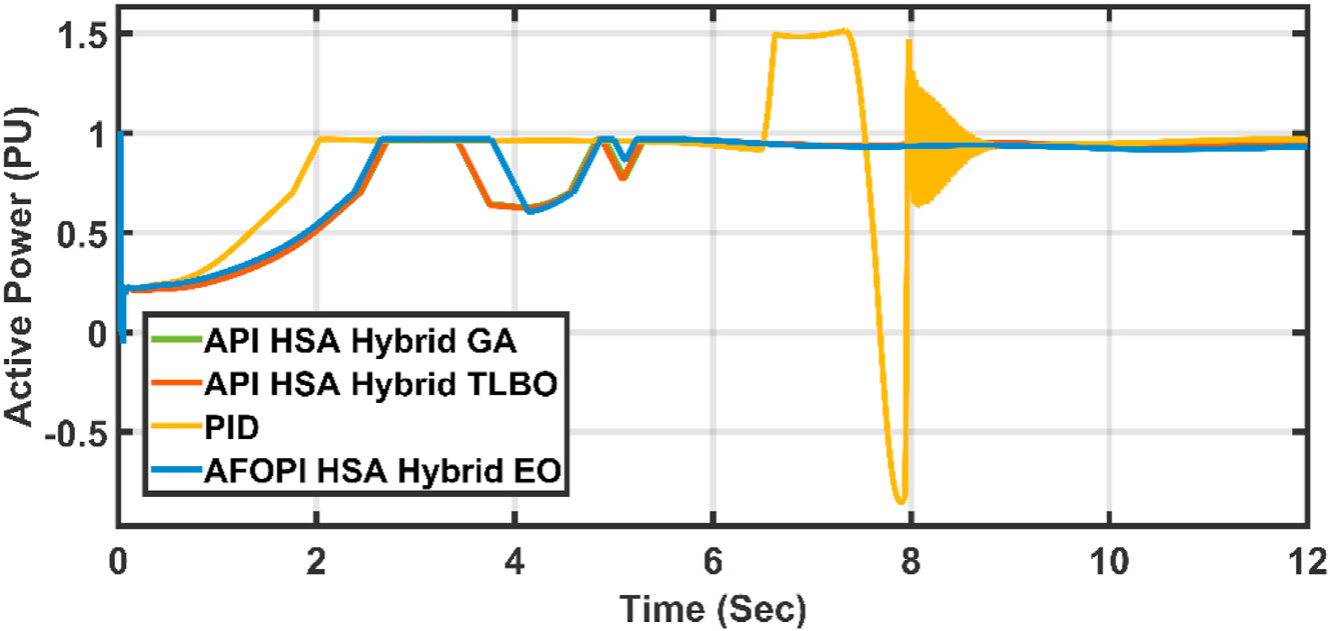
The behavior of the controller active power for normal winter wind speed.

**Figure 14:**
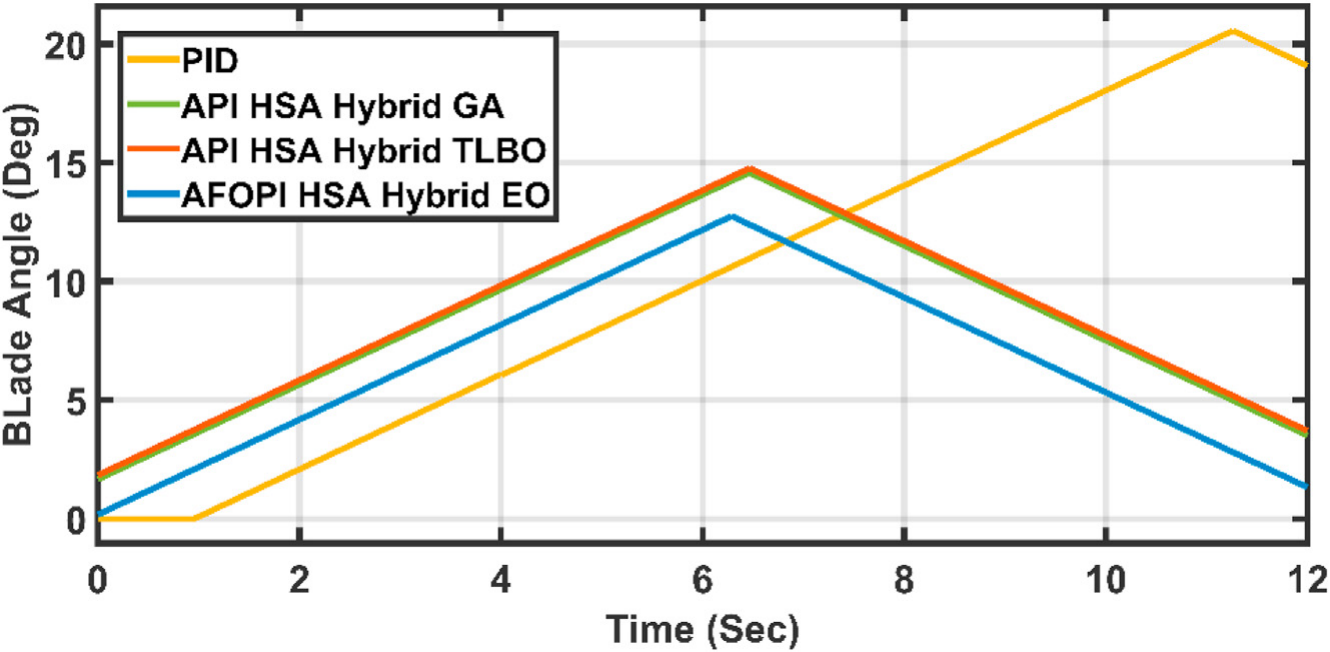
The behavior of controller blade angles for normal winter wind speed.

The active power and the blade angles of the controllers are shown in Figures 13 and 14 respectively. The adaptive controllers show better behavior which are free of oscillations and more stable than the conventional PID. The AFOPI has a shorter rising and settling time than the API. Also, the AFOPI behavior achieves the unity reference rated power value for a longer time than the API. The blade angles of the AFOPI and PID start from zero to extract as much power as possible to decrease the rising time.

The active power statistical analysis of the four controllers is listed in Table 3. The AFOPI HSA-EO has the minimum standard of deviation and the minimum root mean square of error.

**Table 3:** Active power statistical analysis of the four controllers.

Controllers	Standard of deviation (Std)	Root Mean Square of Error (RMSE)
PID HSA	0.4039	0.4406
API HSA-GA	0.3224	0.4303
API HSA-TLBO	0.3245	0.4293
AFOPI HSA-EO	0.3192	0.4176

**Figure 15:**
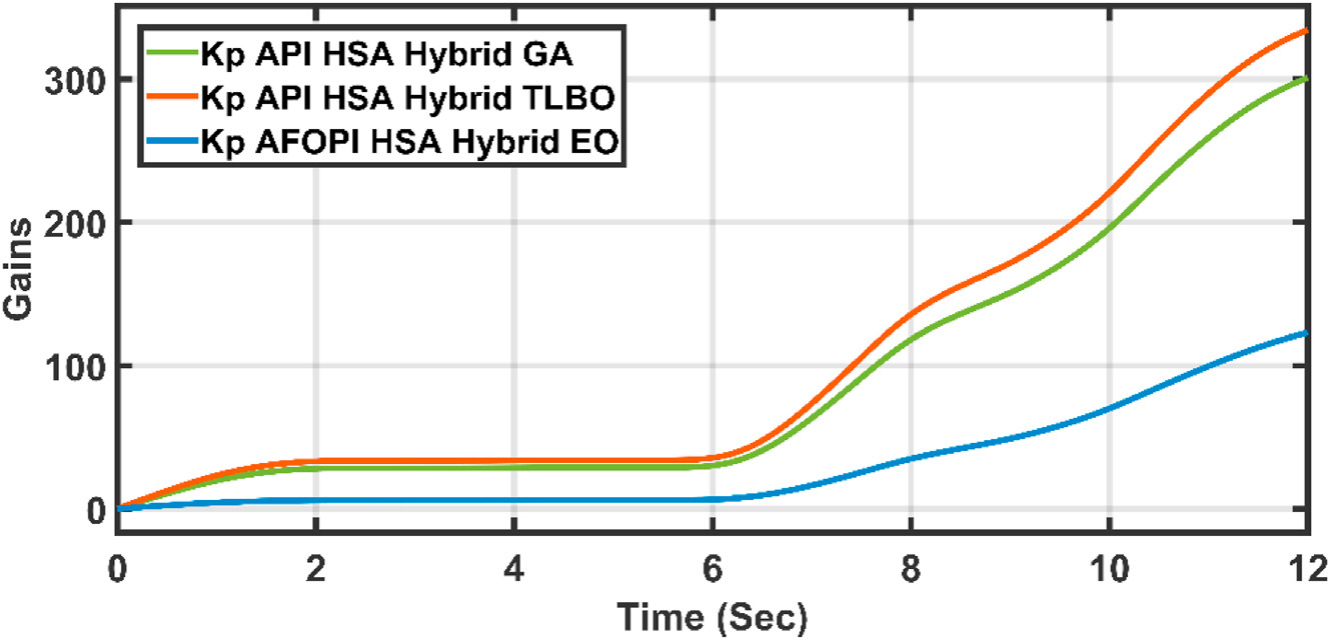
The proportional K_p_ parameters of API and AFOPI controllers.

**Figure 16:**
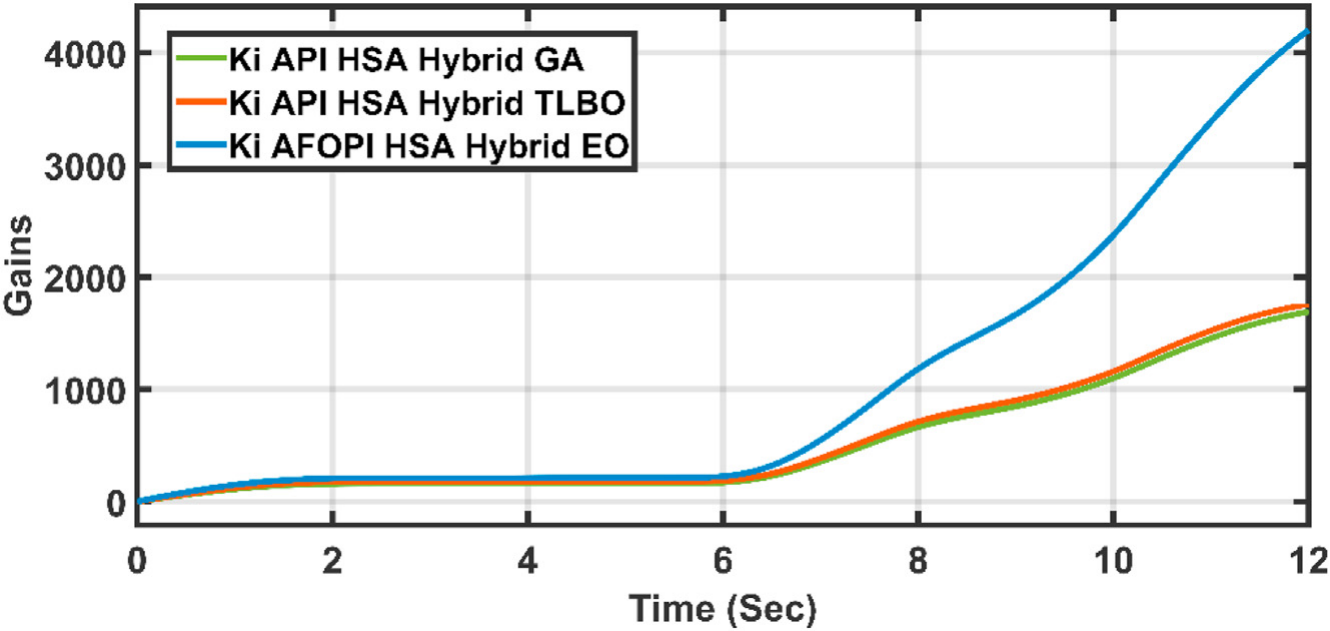
The integral K_i_ parameters of API and AFOPI controllers.

The K_p_ and K_i_ controller parameters are shown in Figures 15 and 16 respectively. The parameters are time-varying, they automatically adjust themselves to withstand the wind speed fluctuations. Because of the fast, continuous variations in wind speed, the parameters are not settled to a constant value.

### 5.2 Validation under faulty conditions

**Figure 17:**
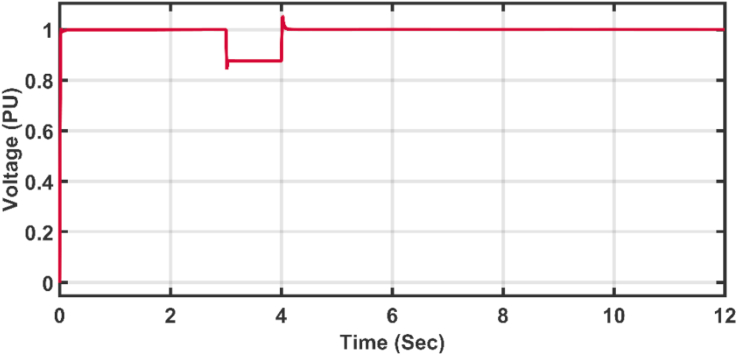
The behavior of the voltage during fault duration.

The same wind speed shown in Figure 12 is again applied to the wind turbine with a faulty scenario. A Line to Ground (LG) short circuit fault is applied to the middle of the transmission line shown in Figure 5. The fault is applied with a fault resistance ($R_{f}$) equal to 0.1 Ω, and the fault lasts for 1 s between the third and fourth seconds as shown in the behavior of the voltage Figure 17. The adaptive PI controller parameters are initialized using the successive trial and error method [32, 33], while the proposed AFOPI controller parameters are initialized using HSA-EO. The parameters of the API and AFOPI are listed in Table 4.

**Table 4:** The controllers parameters.

Controllers	Controller parameters		
	**K**_**c**_	**K**_**1**_	**K**_**2**_
API Trial and Error [32]	90	90	1000
AFOPI HSA-EO	116	71.58	931.034
	**m**	***K***_**3**_	
	0.821	0.9	

**Figure 18:**
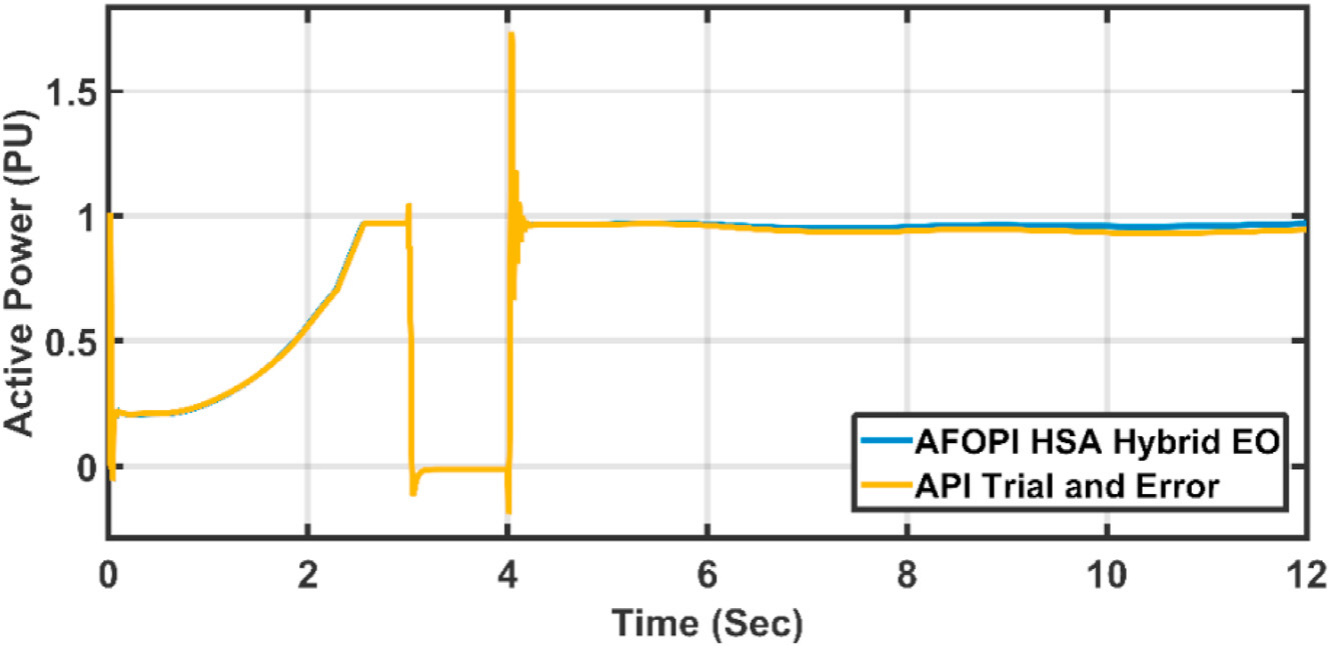
The behavior of the controller active power for faulty winter wind speed.

**Figure 19:**
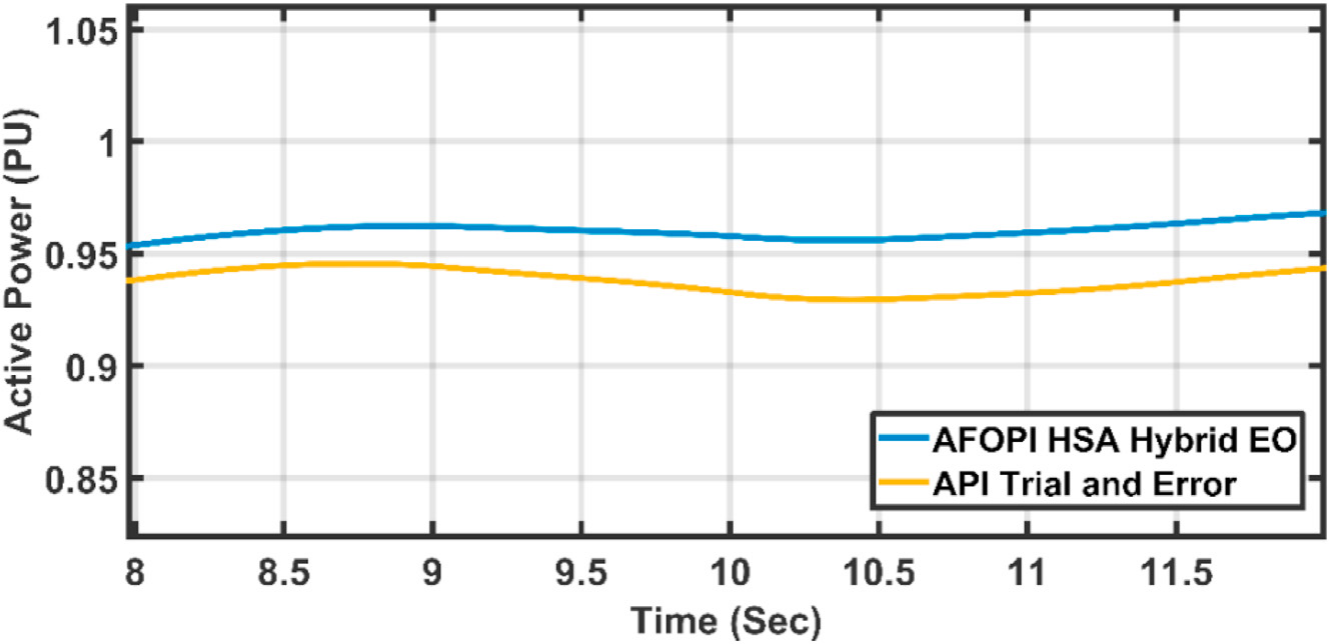
The behavior of the controller active power at focusing view.

The active power of the system is shown in Figure 18. The active power falls during the fault duration and then they succeeded in fault riding through (FRT). Both controllers have nearly the same rising time. Figure 19 shows the last 2 s of focusing view of Figure 18. The steady state error for AFOPI is less than that of the API controller as shown in Figure 19.

The active power statistical analysis of API trial & error and AFOPI HSA-EO is shown in Table 5.

**Table 5:** Active power statistical analysis.

Controllers	Standard of deviation (Std)	Root Mean Square of Error (RMSE)
API Trial and Error	0.4134	0.5328
AFOPI HSA-EO	0.4082	0.4958

As shown in Table 5 the AFOPI HSA-EO has better standard of deviation and better root mean square of error.

**Figure 20:**
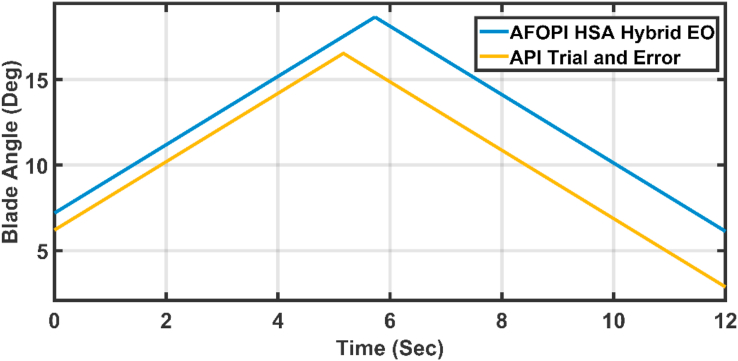
The behaviors of the controller blade angle for faulty winter wind speed.

**Figure 21:**
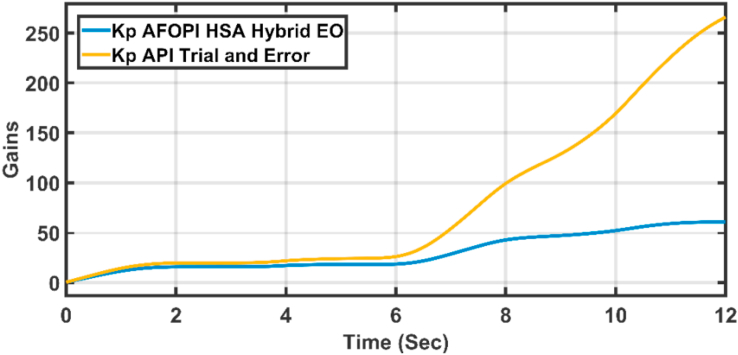
K_p_ parameters of API and AFOPI controllers.

**Figure 22:**
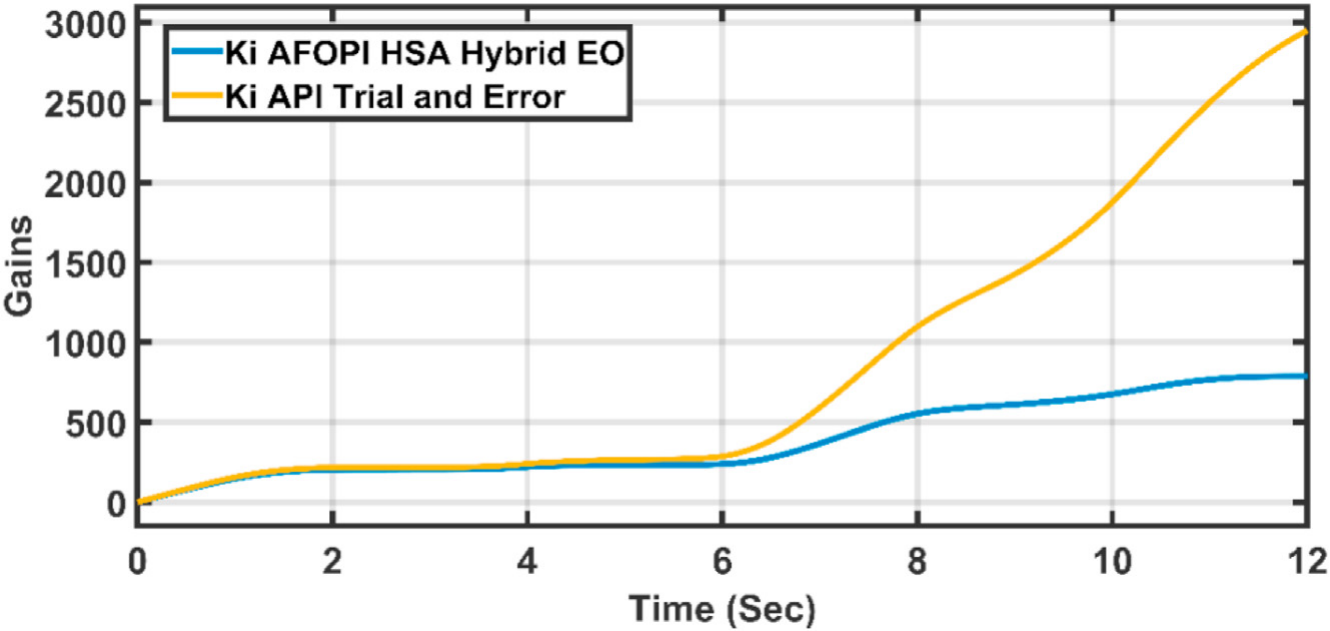
K_i_ parameters of API and AFOPI controllers.

Figure 20 shows the blade angles of the two controllers. While the K_p_ and K_i_ variations of the two controllers are shown in Figures 21 and 22 respectively. Again, the gains are varied to withstand the wind speed fluctuations, while they are nearly constant during the fault 1-s duration. During the fault duration, no active power is produced, and the accumulation of aerodynamic power is converted to rotational kinetic power which causes a sudden increase in the blade's rotational velocity.

## 6 Simulation results

The new proposed controller is subjected to another two case studies to assure its robustness. The controller parameters are kept constant as the values listed in Table 1.

### 6.1 First case study

**Figure 23:**
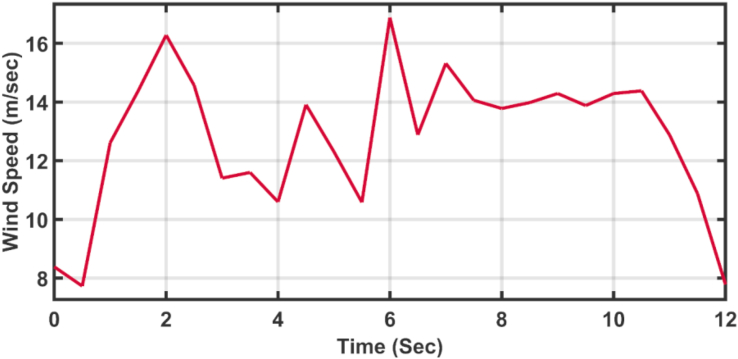
The First case of the applied wind speed profile.

**Figure 24:**
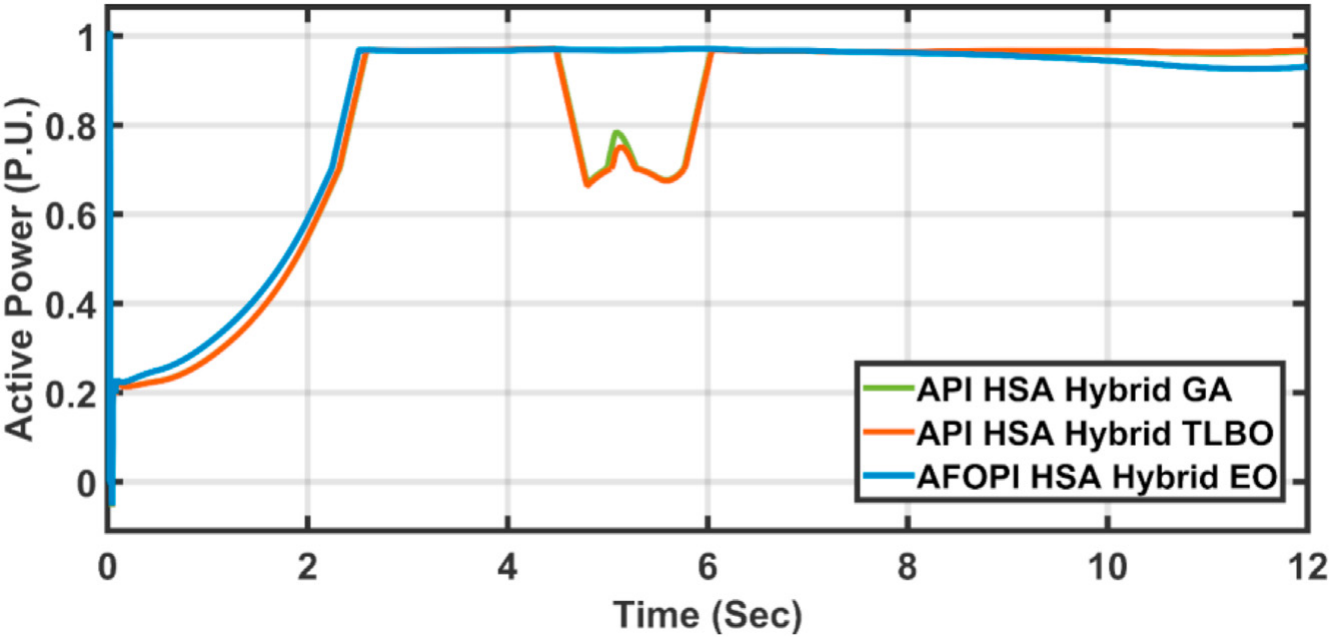
The behavior of the controller active power for the first case study.

**Figure 25:**
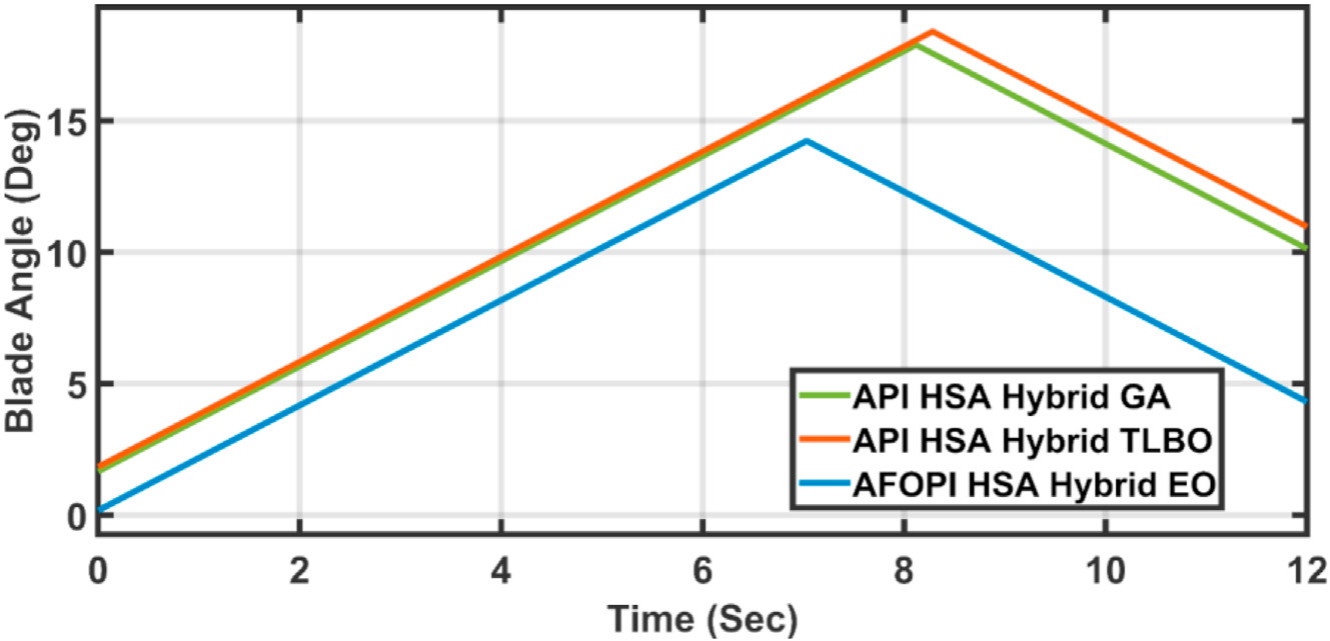
The behavior of the controller blade angle for the first case study.

**Figure 26:**
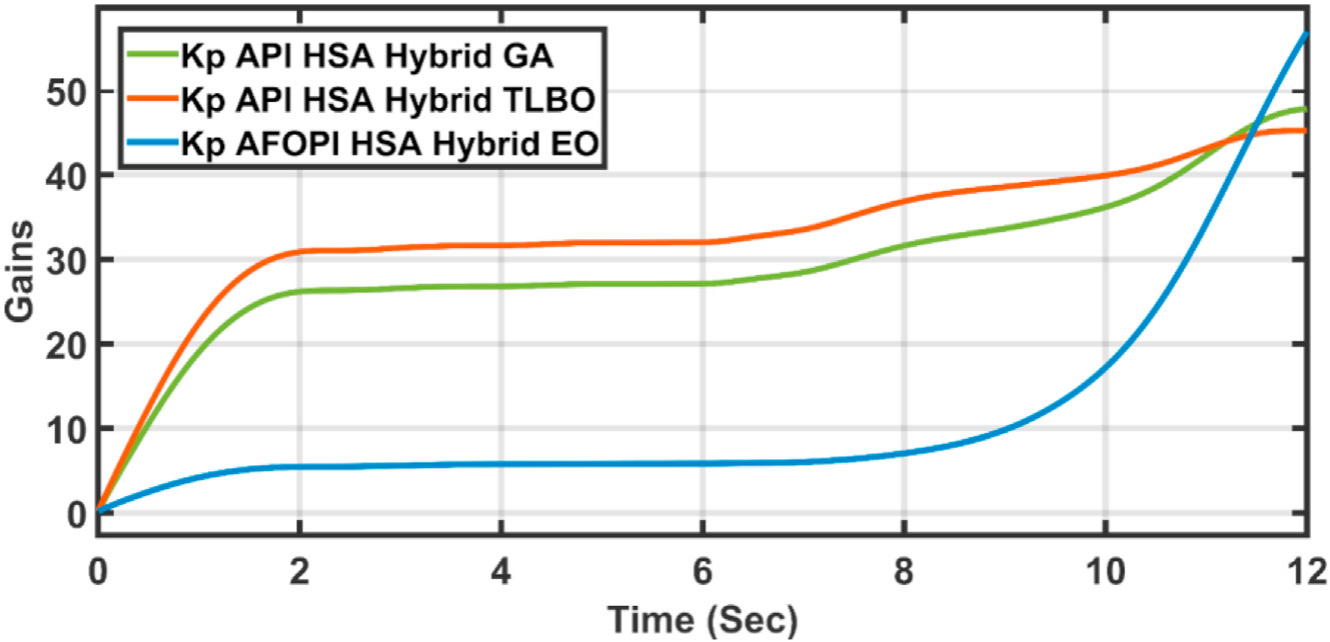
K_p_ parameters of API and AFOPI controllers for the first case study.

**Figure 27:**
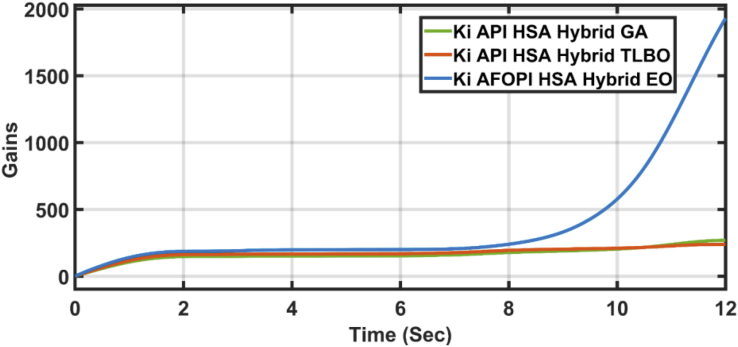
K_*i*_ parameters of API and AFOPI controllers for the first case study.

Figure 23 shows another applied profile of wind speed to the wind turbine. The behavior of the controller active power and blade angle are shown in Figures 24 and 25 respectively. The new proposed AFOPI controller shows a smoother behavior of the active power, with no oscillations and a shorter rising time. The K_p_ and K_i_ controller parameters are shown in Figures 26 and 27 respectively.

The active power statistical analysis of the three controllers is listed in Table 6. The AFOPI HSA-EO has the highest energy captured and the minimum Standard of deviation and the minimum root mean square of error.

**Table 6:** Active power statistical analysis of first case study.

Controllers	Energy	Standard of deviation (Std)	Root Mean Square of Error (RMSE)
API HSA-GA	668.2421	0.3288	0.4218
API HSA-TLBO	681.4193	0.3292	0.4275
AFOPI HSA-EO	683.1449	0.3323	0.4157

### 6.2 Second case study

**Figure 28:**
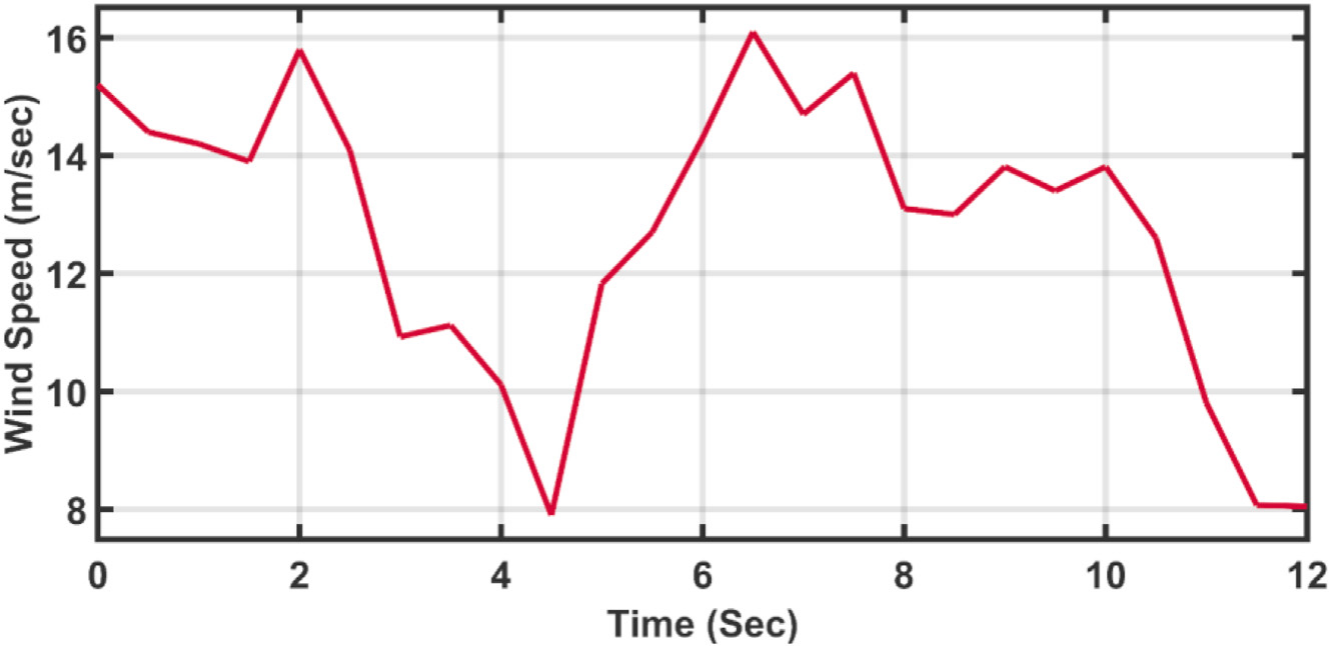
Second case applied wind speed profile.

**Figure 29:**
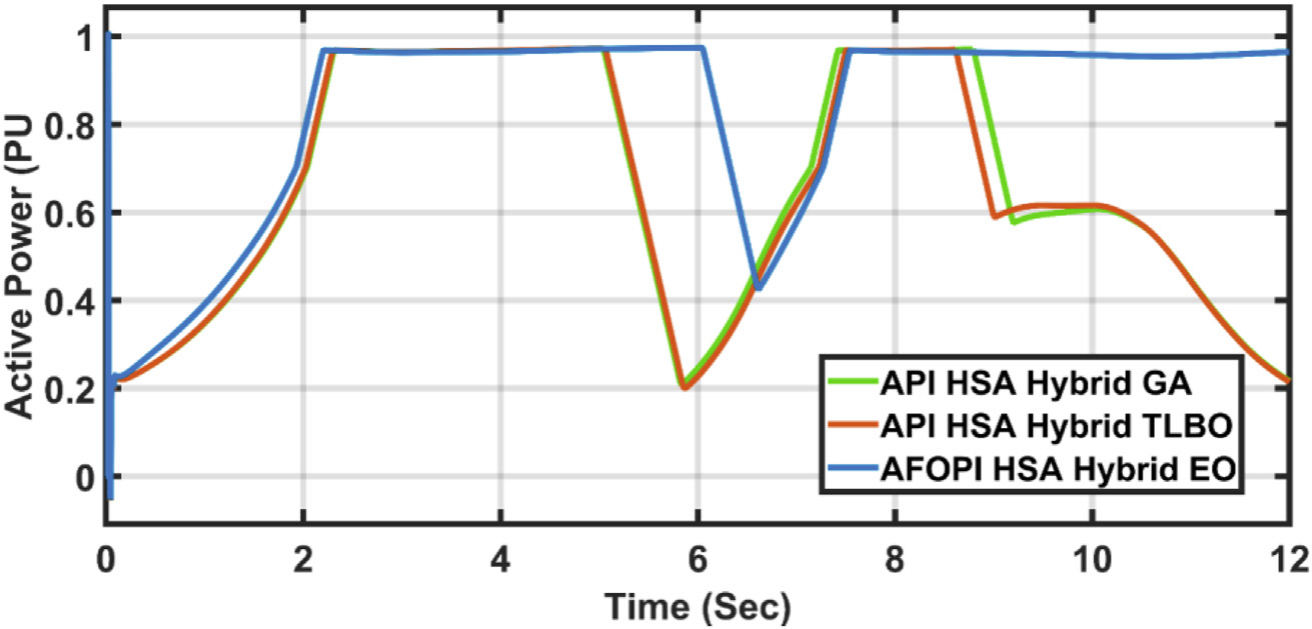
The behavior of the controller active power for the second case study.

**Figure 30:**
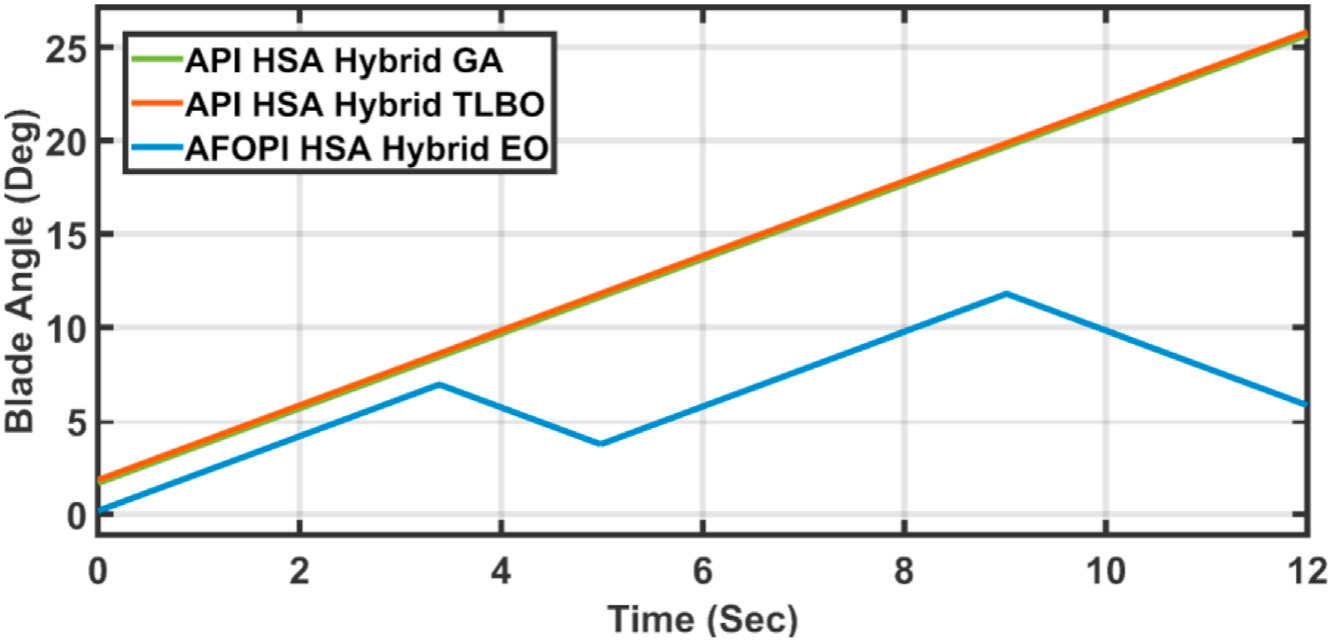
The behavior of the controller blade angle for the second case study.

**Figure 31:**
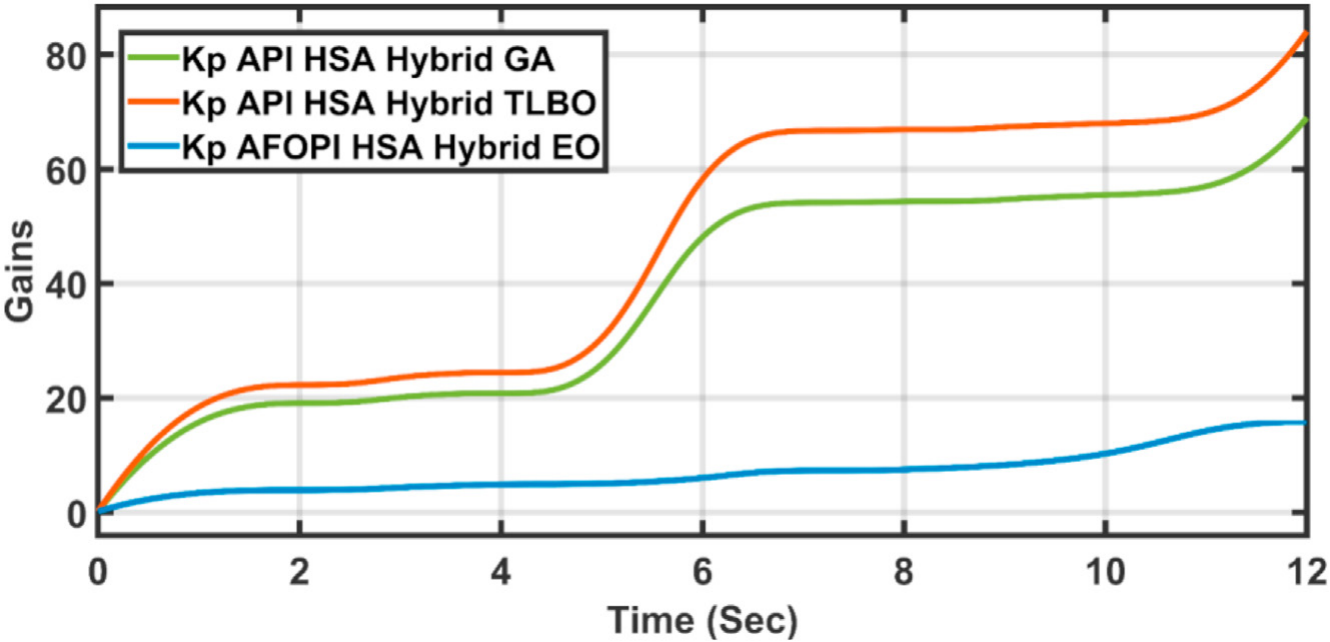
K_p_ parameters of API and AFOPI controllers for the second case study.

**Figure 32:**
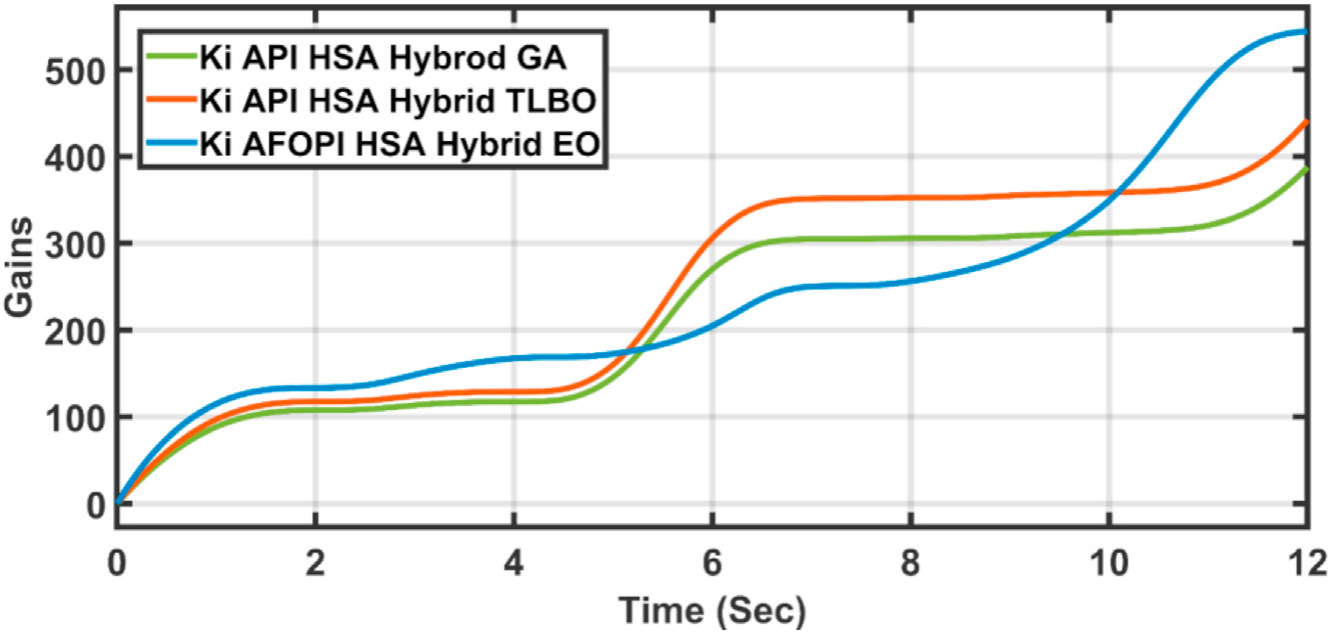
K_i_ parameters of API and AFOPI controllers for the second case study.

Figure 28 depicts another applied gust profile of wind speed to the wind turbine. The behaviors of the controller active power are shown in Figure 29. The new proposed AFOPI controller succeeded in withstanding this gusty wind and keeping the active power at unity value, while the other two controllers (API HSA-GA, API HSA-TLBO) failed. The behaviors of the controller blade angle are shown in Figure 30. The blade angle of the AFOPI has continuous changes to follow up these gust wind speed variations. The K_p_ and K_i_ controller parameters are shown in Figures 31 and 32 respectively.

The active power statistical analysis of the three controllers is listed in Table 7.

**Table 7:** Active power statistical analysis of second case study.

Controllers	Energy	Std	RMSE
API HSA-GA	586.6890	0.3253	0.5197
API HSA-TLBO	557.7517	0.3164	0.5126
AFOPI HSA-EO	729.6914	0.3213	0.3967

## 7 Contribution

The new proposed adaptive fractional order succeeded in increasing the wind farm efficiency. It succeeded in enhancing both the active power and the blade angle under different applied wind speed profiles. Also, the new controller improved the wind farm fault ride through (FRT) capability during the Line-to-ground fault case study.

## 8 Conclusion

The paper presented a new proposed adaptive fractional order PI. The controller acquires the advantage of both: adaptive PI and classical fractional order PI. A validation case study has been carried out to prove the distinction of the new controller with respect to the traditional PID and adaptive PI controllers. The new controller assured its reliability and robustness through surpassing the other controllers with its more stable behavior that is free of oscillations. It also succeeded in making faults ride through with lower steady state errors compared with the conventional adaptive PI. Also, the proposed controller kept the active power extraction under wind speed high variations while the traditional adaptive PI failed. The HSA-EO algorithm is applied to initialize the proposed adaptive fractional order PI controller. The HSA-EO distinction was proved over the HSA-GA and HSA-TLBO.

## Declarations

### Author contribution statement

Ahmed M. Shawqran & Mahmoud A. Attia: Conceived and designed the experiments; Performed the experiments; Wrote the paper.

Abdallah El-Marhomy, Almoataz Y. Abdelaziz & Hassan Haes Alhelou: Analyzed and interpreted the data; Contributed reagents, materials, analysis tools or data.

### Funding statement

This research did not receive any specific grant from funding agencies in the public, commercial, or not-for-profit sectors.

### Data availability statement

Data will be made available on request.

### Declaration of interests statement

The authors declare no conflict of interest.

### Additional information

No additional information is available for this paper.
